# Comparative myogenesis in teleosts and mammals

**DOI:** 10.1007/s00018-014-1604-5

**Published:** 2014-03-25

**Authors:** Giuliana Rossi, Graziella Messina

**Affiliations:** Department of Biosciences, University of Milan, 20133 Milan, Italy

**Keywords:** Mouse and zebrafish, Myotome and dermomyotome, Primary and secondary myogenesis, Muscle fiber types, Satellite cells, Regeneration

## Abstract

Skeletal myogenesis has been and is currently under extensive study in both mammals and teleosts, with the latter providing a good model for skeletal myogenesis because of their flexible and conserved genome. Parallel investigations of muscle studies using both these models have strongly accelerated the advances in the field. However, when transferring the knowledge from one model to the other, it is important to take into account both their similarities and differences. The main difficulties in comparing mammals and teleosts arise from their different temporal development. Conserved aspects can be seen for muscle developmental origin and segmentation, and for the presence of multiple myogenic waves. Among the divergences, many fish have an indeterminate growth capacity throughout their entire life span, which is absent in mammals, thus implying different post-natal growth mechanisms. This review covers the current state of the art on myogenesis, with a focus on the most conserved and divergent aspects between mammals and teleosts.

## Introduction

Skeletal muscle is the most abundant tissue in vertebrates, and it is used for locomotion, breathing, and energy metabolism. Different muscles have distinct features, including anatomical structure, contractile and metabolic properties, fiber composition, blood supply, pattern of innervation, and embryonic origin. In addition, different muscles have different regenerative capacities [1] and are differentially affected in genetic disorders [2]. All the muscles of the limbs and trunk originate from the somites [3], whereas for the head, only the muscles of the tongue and some of the larynx and neck muscles are believed to be of somitic origin [4].

The fundamental events in myogenesis that are common to all vertebrates are the specification of the progenitor cells according to myogenic lineage, proliferation and migration, cell-cycle exit, differentiation, and fusion. The transcription factors (or myogenic regulatory factors, MRFs) that are responsible for the commitment of mesodermal cells to a muscle lineage (i.e., MyoD, Myf5) and for the initiation and maintenance of the terminal differentiation program (i.e., Myogenin, Mrf4) are highly conserved in teleosts and mammals [5]. Teleosts, and in particular zebrafish (*Danio rerio*), are useful for practical reasons, including ease of genetic manipulation and the large number and optical clarity of embryos/larvae that can be obtained, which allows cell movements to be observed in real time. Skeletal muscle development in teleosts shares several common features with that observed in amniotes: multistep development that involves the appearance of different classes of progenitor cells, the formation of myotome/dermomyotome, the molecular signals that drive commitment and differentiation, and the presence of muscle fibers with different contraction properties [6–8]. Nevertheless, myogenesis has some unique features in teleosts compared to mammals, which include the early stage of muscle commitment, presence of adaxial cells, different proportions of slow and fast fibers, and muscle growth throughout much of ontogeny. Moreover, on the basis of their different development, the main phases of myogenesis in teleosts consist of the embryonic–larval–juvenile and adult stages, whereas mammalian muscle development is conventionally divided into pre-natal and post-natal, and thus it is not always possible to make direct comparisons. In both mammals and teleosts, muscle development occurs through distinct myogenic waves that will be reported and discussed in this review, which will particularly focus on the conserved and divergent aspects between mammals and teleosts. Although we have tried to be as complete as possible, the wide topic covered prevents a full discussion of the original reports on which current knowledge in this field is based. Therefore, readers will also be referred to recent reviews and articles that cover specific aspects.

## Embryonic myogenesis

### Prenatal muscle development in mammals

#### Anatomical structures

During embryonic myogenesis, mesoderm-derived somites generate the first muscle fibers of the body, and, in subsequent waves, additional fibers are generated along the first muscular template [9]. *Somites* are transient mesodermal units that develop in a cranial to caudal succession from the segmental plate of the paraxial mesoderm [3]. As development proceeds, somites form into the distinct structures of a ventral sclerotome and a dorsal *dermomyotome*, which becomes the source of myogenic progenitors. Shortly after the onset of somitogenesis (at embryonic day E8.75 in mouse), some myogenic precursors give rise to terminally differentiated, mononucleated muscle cells (myocytes) of the primary *myotome*. The development of the primary myotome is a process in which precursors translocate from the dermomyotome to a ventrally located domain where they elongate along the axis of the embryo. This process has been widely studied, in particular in the avian embryo, although the role of the myotome during development of mammals remains unclear [4, 10–17]. In particular, in Myf5^nLacZ/nLacZ^ mice, in which both Myf5 and Mrf4 expression is abolished, the primary myotome fails to form, whereas myogenesis proceeds normally, which would suggest that the myotome is dispensable for later muscle development [18]. In the dermomyotome, two regions can be further distinguished on the basis of their distance from the neural tube, and these give rise to the *epaxial and hypaxial musculature*. The epaxial dermomyotome is located dorsally and leads to the deep muscles of the back in amniotes, whereas the hypaxial dermomyotome is located superficially, laterally, and ventrally, and gives rise to the diaphragm, body wall, and limb muscles [19]. Evidence from several studies has demonstrated that the epaxial myogenic progenitors are dependent upon signals from axial structures, such as Sonic Hedgehog (Shh) and Wingless 1 (Wnt1), which mainly activate a myogenic program through the induction of Myf5. In contrast, hypaxial progenitors require signals from the dorsal ectoderm, such as Wnt7a, to promote MyoD-dependent myogenesis [19, 20]. This is consistent with the phenotype observed in the *Myf5* and *MyoD* knock-out embryos, in which the former have epaxial defects and the latter show a delay in limb myogenesis, although the other myogenic determinant genes can drive an almost normal skeletal muscle development, as also explained by the primaxial–abaxial theory [21–23].

In mouse, the roles and interplay among the MRFs have been widely studied. Myf5 and MyoD have a largely redundant function in myoblast determination, so that deletion of the *Myf5* or *MyoD* genes does not significantly affect muscle development [22, 24], but deletion of both *Myf5* and *MyoD* eliminates skeletal muscle lineage [25]. Of note, it has been demonstrated that Mrf4 is also involved in mouse muscle determination, as *Myf5:MyoD* double-mutant mice are actually partial triple mutants, because the deletion of the *Myf5* locus also compromises the genetically linked *Mrf4* gene expression [26]. Indeed, in mutant embryos in which Mrf4 expression is preserved, embryonic myogenesis takes place in the absence of *MyoD* and *Myf5*, even if the muscle rapidly degenerates into the fetal stage of development [26]. This is in agreement with previous observations, which have shown that Mrf4 is transiently expressed during somitogenesis and later during fiber maturation [27]. Remarkably, the mouse *Myogenin* gene acts genetically downstream of *Myf5* and *MyoD* to drive committed myoblasts towards terminal, biochemical muscle differentiation; if *Myogenin* is absent, myoblasts are correctly specified and positioned, but they fail to differentiate [28, 29]. Although Mrf4 is not essential for later muscle development, *Mrf4:MyoD* double-mutant mice are phenotypically similar to *Myogenin* mutants, which indicates that Mrf4 and MyoD have redundant roles in the activation of the differentiation program [30].

**Fig. 1:**
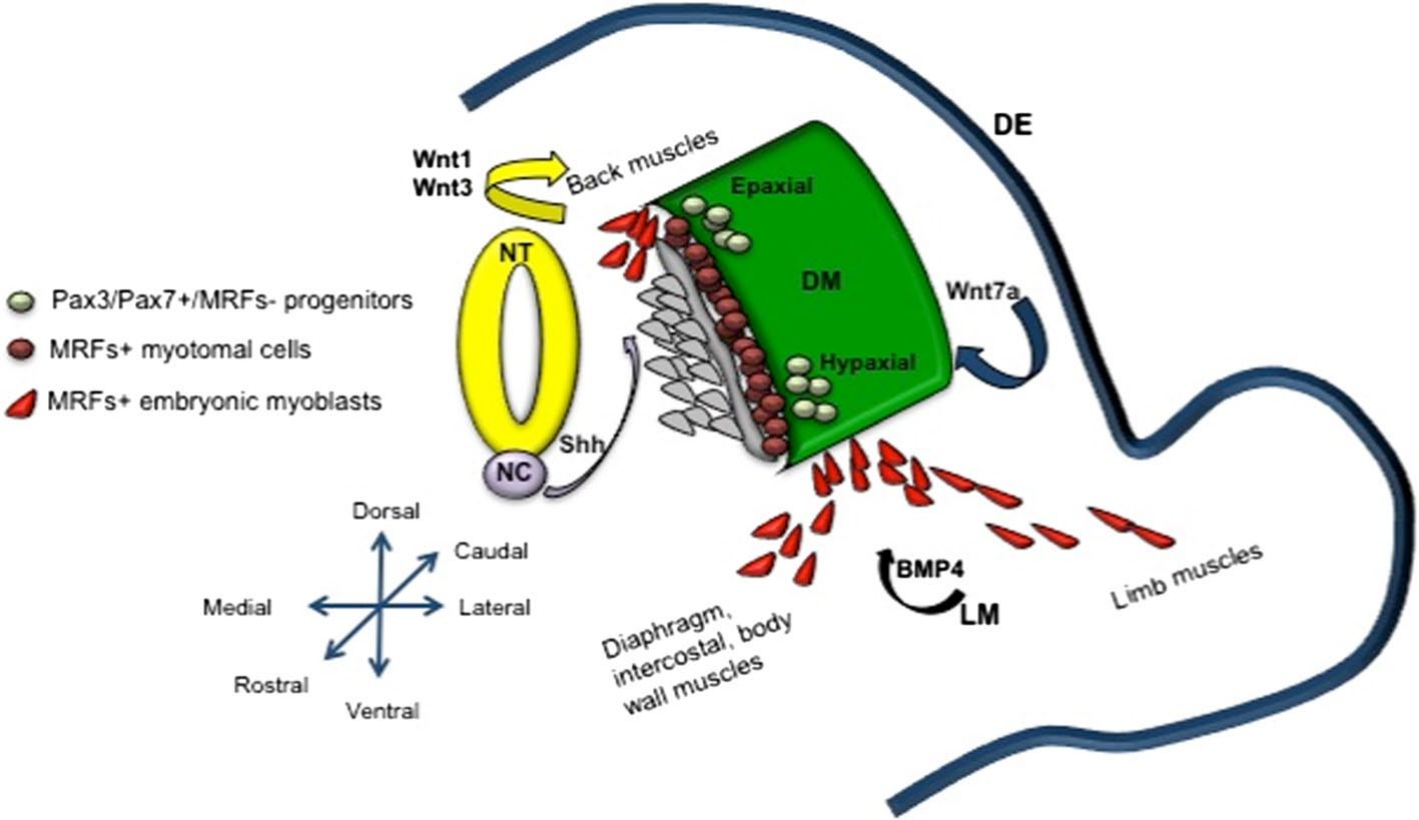
Model of the early phases of myogenesis in mouse at embryonic day E10.5, illustrating how morphogens secreted by the surrounding domains can influence myogenic commitment. *DM* dermomyotome, *DE* dorsal ectoderm, *NT* neural tube, *NC* notochord, *LM* lateral mesoderm, *MRFs* myogenic regulatory factors

A broad spectrum of signaling molecules drives myogenesis during embryonic development [31]. These include morphogens that converge and act on a battery of transcription and chromatin-remodeling factors, which in turn drive cell progenitors towards a myogenic fate. On the basis of the concentration and the distance from the source, morphogens lead to different cellular fates [32]. As indicated above, Wnts and Shh are strongly involved in the positive specification of muscle progenitors in the somite. Mouse mutants for Wnt1, and the functionally redundant Wnt3a, have dermomyotome defects and reduced expression of the paired-homeobox transcription factor Pax3, as well as Myf5 [33]. Different findings in the literature show that Shh is essential for the commitment of dermomyotomal cells in MyoD/Myf5-positive myoblasts [34–36]. In contrast, bone morphogenic proteins (BMPs), which are members of the TGF-β superfamily, have opposite effects on the myogenic program. In particular, Bmp4 is expressed in the lateral-plate mesoderm, and it retains muscle progenitors in an undifferentiated state by inducing Pax3 expression, and thus delaying Myf5 and MyoD induction [37] (Fig. 1).

Notch signaling has been described as critical in the fate decisions of progenitor cells [38]. Notch mediates cell–cell communication, and has been described to inhibit myogenesis through inhibition of MyoD in cooperation with the DNA-binding protein RPB-J and the transcriptional repressor Hes1 [39, 40]. In particular, mutations in the Notch ligand *Delta1* or in *RPB*-*J* lead to precocious and robust muscle differentiation and loss of muscle precursors [41]. This suggests that, as for BMP4, Notch signaling promotes the expansion of myogenic progenitors while preventing differentiation.

**Fig. 2:**
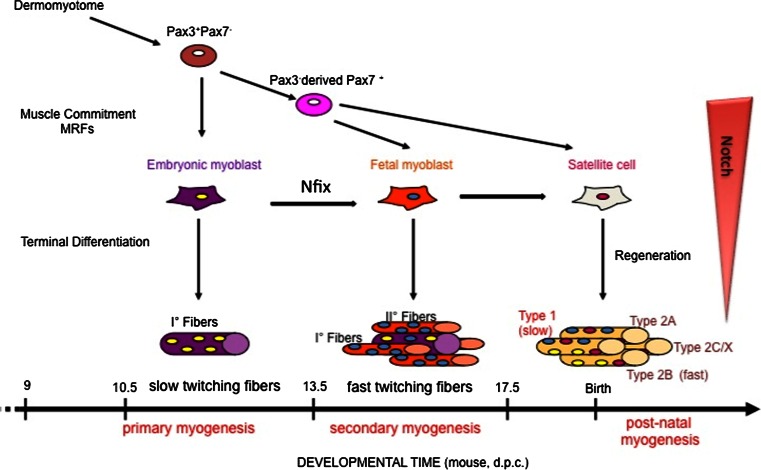
Scheme of myogenic lineages, myogenic waves, and the molecules responsible for pre-natal muscle development in mouse. *MRFs* myogenic regulatory factors, *d.p.c.* days post-coitum. An indicative timing of murine development is shown

The next level in the hierarchy of the control of myogenesis has as its major players the paired-homeobox transcription factors Pax3 and Pax7. All vertebrates have one of these genes, and it has been suggested that their evolutionary origin arose from the duplication of a common ancestral gene [42]. Pax3 and Pax7 are expressed in the dermomyotome, with the highest levels of Pax3 in the dorsal and ventral lips, and of Pax7 in the central domain [43]. Both Pax3 and Pax7 depend on the expression of sine-oculis-related homeobox 1 (Six1) and Six4, which are currently considered to be at the apex of the genetic regulatory cascade that directs dermomyotomal progenitors towards the myogenic lineage [44, 45]. In contrast to chick embryos, where the migrating progenitor cells already express Pax7, in mouse, myogenic progenitors start to express Pax7 only when they have already entered the limbs [46]. Indeed, in mouse, it is Pax3 alone that is required for delamination and migration of somatic precursor cells into the limb buds, through the signaling that involves scatter factor/hepatocyte growth factor (SF/HGF) and its receptor, the tyrosine kinase c-Met, which is a direct target of Pax3 [47] (Fig. 2). Mouse embryos that are homozygous for the Splotch *Pax3* loss-of-function mutation do not develop the hypaxial domain of the dermomyotome, and, consequently, the limb and diaphragm muscles do not form [48–50]. Pax3 acts genetically upstream of MyoD, as no MyoD transcript can be detected in the limbs of Splotch mutant embryos. In particular, *Pax3:Myf5:Mrf4* triple mutants have a dramatic phenotype that is not seen for the individual mutants: the body muscles are absent. MyoD does not rescue this triple-mutant phenotype, as activation of *MyoD* has been shown to be dependent on either Pax3 or Myf5 [51]. On the other hand, Pax7 appears to be dispensable during embryonic muscle development [52]. Of note, muscle development is more defective in the *Pax3:Pax7* double knock-out, in which further muscle development is arrested and only the early embryonic muscle of the myotome is formed [53]. In addition, when Pax7 is knocked-into the Pax3 locus, most of the functions of Pax3 are restored [54]. A more defined role for Pax3-expressing and Pax7-expressing myogenic populations was well described by the group of Kardon, and will be further discussed in the next section [55].

The *head musculature* has a different scenario, as it develops through different mechanisms. The craniofacial muscles have always been considered to be intricate muscles, and they have been less well explored. In recent years, remarkable progress has been made that has clearly revealed new concepts that have cast doubt on some of the classical dogma. Craniofacial skeletal muscles can be divided into distinct classes, and at variance with muscles of the trunk and limbs, these classes are not all of somitic origin: somite-derived tongue and neck muscles; extraocular muscles that originate from the cranial paraxial and prechordal mesoderm; and branchiomeric muscles that are derived from the cranial mesoderm which is transiently present in the pharyngeal arches [56–58]. The first indication that the head muscles develop differentially from the muscles of the trunk and limbs came from the observation that murine *Pax3:Myf5(Mrf4)* mutants cannot form body muscles, whereas the head muscles are present [51]. In addition, signaling that has been widely described as promoting myogenesis in the trunk and limbs, such as the Wnt and BMP pathways, has inhibitory effects in the head [59, 60]. Interestingly, Pax3, which has a fundamental role in trunk and limb muscle development [48, 53, 54], is not expressed in head muscles, and no muscular defects have been reported in the head for *Pax3* mutant mice. Another fascinating difference is that Mrf4, which is important for myogenic determination of limb and trunk progenitors, cannot fulfil the same role in the head [26]. It is now known that all the head muscles depend on Pitx2 and Tbx1, which are transcription factors that contain homeodomains, and which positively regulate one another as well as Myf5; Pitx2 and Tbx1 thus regulate the myogenic cascade [61–63]. Recently, it has been shown that only the extraocular eye muscle, and not other head muscles, depends on the presence of both Myf5 and Mrf4, whereby MyoD cannot compensate for their absence [64]. In contrast, Tbx1 and Myf5 are necessary for branchiomeric muscle differentiation that converges on MyoD, as in trunk myogenesis. Of note, a contribution to skeletal muscle development of the lateral plate mesoderm in chick and mouse has recently been demonstrated, where it has been shown that the somatopleure that is adjacent to the first three somites contributes to the development of some of the neck muscles [65].

#### The different classes of myogenic populations in mammals

It has been widely demonstrated in mammals that, like hematopoiesis, skeletal myogenesis occurs in successive, distinct-though-overlapping developmental stages that involve different cell populations and the expression of different genes. Skeletal muscle is, indeed, a heterogeneous tissue that is composed of individual muscle fibers that are diverse in size, shape, and contractile protein content, through which they can fulfill the different functional needs of the vertebrate body. This heterogeneity derives and depends at least in part upon distinct classes of myogenic progenitors; i.e., embryonic and fetal myoblasts and satellite cells (SCs). In particular, embryonic and fetal myoblasts control differentiation of the pre-natal musculature, whereas SCs are responsible for post-natal muscle growth and regeneration following muscle damage or injury [66–68]. Myoblast fusion into multinucleate muscle fibers begins at around E11 in mouse, and it characterizes the ‘embryonic’ or primary myogenesis that is necessary to establish the basic muscle pattern. Fetal myogenesis is characterized by growth and maturation of each muscle anlagen and by the onset of innervation. This second wave of myogenesis (also called secondary myogenesis) takes place between E14.5 and E17.5 in mouse, and it involves the fusion of fetal myoblasts either with each other, to form secondary fibers (initially smaller and surrounding primary fibers), or to a minor extent, with primary fibers. At the end of this phase, a newly formed basal lamina surrounds each individual fiber, and the SCs can then be morphologically identified as mononucleated cells that lie between the basal lamina and the myofiber plasma membrane. Embryonic and fetal cells and SCs were initially classified based on their in vitro characteristics. They differ in terms of their time of appearance, media requirements, response to extrinsic signaling molecules, drug sensitivity, and morphology of the myofibers they generate [66, 69]. In addition, primary, secondary, and adult myofibers differ in their muscle contractile proteins, including their myosin heavy chain (MyHC) isoforms [70–72]. A genome-wide expression analysis carried out on purified embryonic and fetal myoblasts [67] identified many differentially expressed genes, which clearly shows that embryonic and fetal myoblasts are intrinsically different populations of myoblasts with distinct genetic programs.

We have demonstrated the pivotal role of the transcription factor nuclear factor IX, Nfix, in driving the transcriptional switch from embryonic to fetal myogenesis, and therefore from slow muscle to fast twitching and more mature muscle [73]. Nuclear factor one (NFI) proteins function as transcriptional activators and/or repressors of cellular and viral genes. In vertebrates, the *Nfi* gene family consists of four closely related genes, known as *Nfia*, *Nfib*, *Nfic*, and *Nfix*, the last of which is the most expressed in muscle [74]. These encode for proteins with conserved N-terminal DNA-binding and dimerization domains and C-terminal transactivation/repression domains. In vitro and in vivo loss-of-function (using siNfix in fetal myoblasts) and gain-of-function (expression of the exogenous Nfix2 isoform in embryonic myoblasts) approaches have revealed the crucial role of Nfix in driving the transcriptional switch from embryonic to fetal myogenesis. In particular, we showed that Nfix, the expression of which in fetal muscle is in part activated by Pax7, can act through different pathways to switch off embryonic specific markers, such as slowMyHC (by down-regulation of the slowMyHC activator NFATc4), and to activate fetal-specific proteins, such as β-enolase and MCK [73]. Our study thus provided the first evidence that a single factor is responsible for the differential gene expression that transforms the primary muscle anlagen (due to embryonic myogenesis) into a more mature and organized muscle (fetal myogenesis) [73].

Although these myoblast classes are functionally distinct, it was not clear whether they develop from common or different progenitors. As indicated above, Pax3 and Pax7 are markers for somitic myogenic precursors and are crucial for myogenic determination. In a very elegant study, the group of Kardon used *Pax3*-cre and *Pax7*-cre diphtheria toxin mouse strains to clearly demonstrate the outcome of specific ablation of these respective cell populations [55]. Here, Hutcheson et al. clearly demonstrated that loss of the Pax3 lineage is embryonically lethal and prevents the emergence of Pax7-positive cells, whereas ablation of Pax7-expressing cells only leads to defects in the later stages of development, which leads to smaller muscles with fewer myofibers at birth. Thus, they clearly defined Pax3-positive cells as the progenitors of embryonic myoblasts that lead to the development of primary fibers in the limb, to which Pax7-positive cells successively contribute by forming secondary fibers and establishing the SC pool [55].

Although these and other studies have established the origins and features of these myogenic populations, it is still not clear whether there is a single self-renewing population in the embryo that is sustained to adulthood, or whether intermediate, stage-specific populations arise that lead to the populations of the corresponding developmental stages. A recent study supports the former scenario. Using different genetic tools, the group of Tajbakhsh demonstrated that Notch signaling via the Rbpj-dependent pathway is active throughout development in muscle founder cell populations [75]. Notch activity is necessary and sufficient for the maintenance of muscle progenitor cells, and it allows them to still be receptive to specification and differentiation cues during development. Specifically, during embryonic myogenesis, the upstream myogenic subpopulation that expresses high levels of Pax7 (referred to as Pax7^High^) is maintained and expanded by high Notch activity, and their following differentiation is induced by the MRFs, which results in down-regulation of Notch and Pax7 and the formation of myofibers of the corresponding developmental stage. Remarkably, although they are correctly committed, myoblasts with high Notch activity fail to terminally differentiate in embryonic and fetal trunk and limbs, and also in the head (Fig. 2).

#### The heterogeneity of primary and secondary fibers

As indicated above, muscle fiber formation in vertebrates is a multiphasic process that is characterized by heterogeneous fiber types, on the basis of the expression of the different MyHC isoforms. The classification is based on the speed of contraction of the muscle fibers, which mainly depends on the ATPase activity. In rodents, a single I/β slow *MyHC* gene has been identified, the gene product of which is characterized by slow ATPase activity, whereas the embryonic and perinatal MyHC isoforms are progressively replaced post-natally with the three adult fast MyHCs, IIa, IIx, and IIb [76]. The boundaries between the different classes of fibers are not absolute and intermediate fibers can co-express different MyHC isoforms. In particular, during pre-natal muscle development, primary muscle fibers express embryonic (fast) and I/β slowMyHC. In contrast, secondary muscle fibers express the fast embryonic and perinatal isoforms from their inception, and, with the exception of the *soleus* muscle, they do not express the I/β slowMyHC. Thus, in general, mammalian primary muscle fibers are programmed for a mainly slow phenotype, whereas secondary muscle fibers adopt a fast phenotype [66, 67]. This diversification of muscle fibers starts during the embryonic stages, independently of neural influence, whereas, during early post-natal development and in the adult, motor neuron and the activities of various hormones can modulate the fiber type profile, and in particular that of thyroid hormone. In recent years, many key factors that control muscle fiber-specific gene programs have been identified, such as the NFAT isoforms [77]. In addition, Nfix indirectly represses slowMyHC expression through direct inhibition of the *NFATc4* promoter [73].

However, the molecular and cellular mechanisms by which muscle fiber diversity is achieved during development are still poorly understood. Sox6 belongs to the group D of Sox factors, and it is a transcriptional repressor that has been shown to have an important role in the development of several tissues, including skeletal muscle [78]. Different studies have demonstrated that *Sox6*-null muscle has increased levels of I/β slowMyHC and a general switch towards a slower phenotype [79–81]. It has been shown that Sox6 exerts its function by direct binding to the *I/β slowMyHC* promoter [79, 80, 82]. Moreover, the group of Olson identified a microRNA (miR)-mediated transcriptional regulatory network. Here, miR-499 and miR-208 are intronically encoded within the *slowMyHC* genes, and they act through a reciprocal negative-feedback loop to target Sox6, which thus promotes a fast-to-slow myofiber-type switch [81]. As Sox6 does not have any known regulatory domains, the specific mechanisms of repression involved here remain to be elucidated.

In mouse embryo, it has also been demonstrated that the Six1 and Six4 homeodomain transcription factors are required for the correct transcription of fast muscle genes in the myotome, as, in *Six1:Six4* double mutants, the slow program is activated and the fast muscle genes are not expressed [83].

### Embryonic myogenic development in zebrafish

#### Somite development and embryonic myogenesis

In teleosts, the overall process of somitogenesis is similar to mammals, but the timing and the specification of myogenic progenitors show particular differences.

**Fig. 3:**
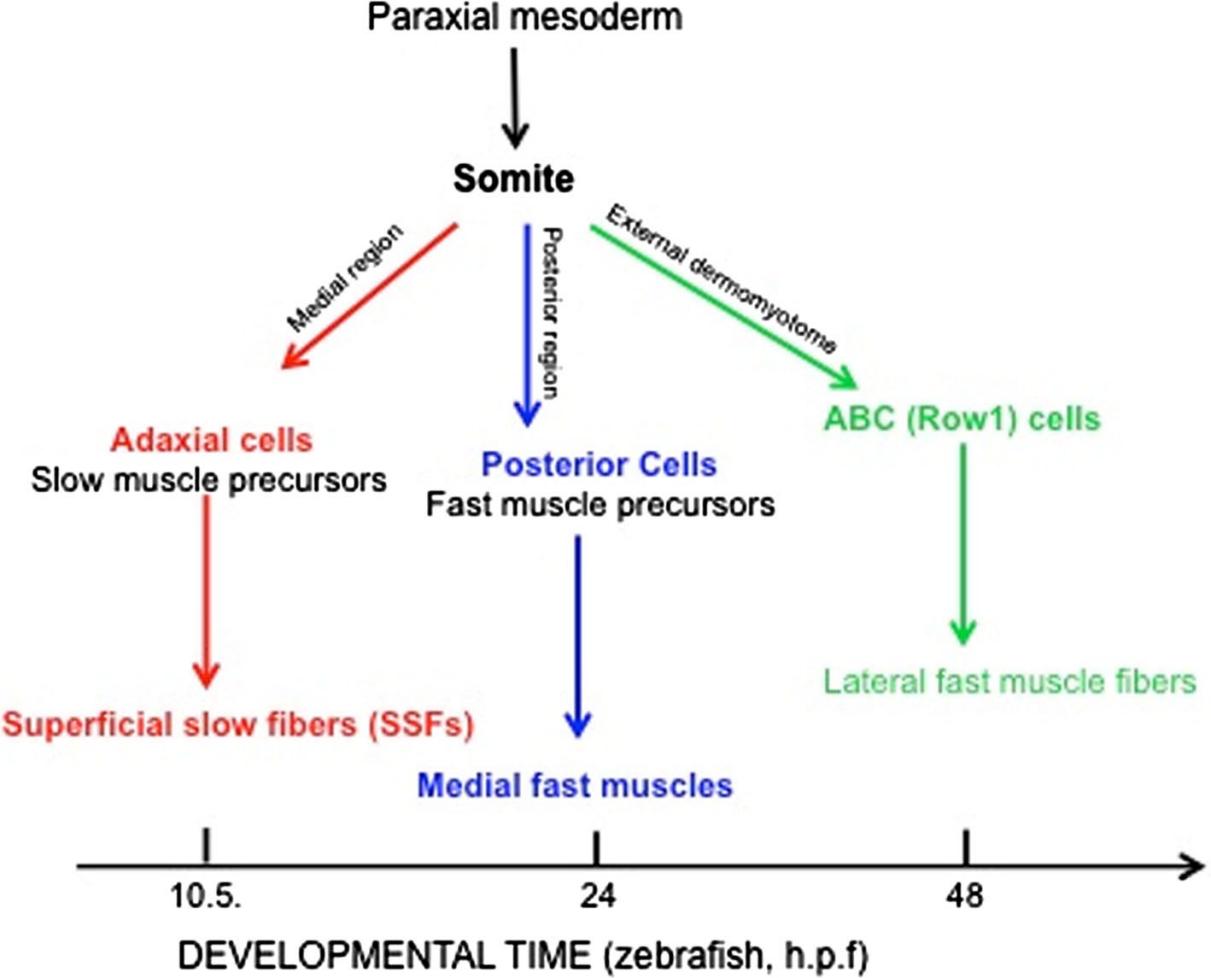
Scheme of embryonic muscle development in zebrafish, showing a schematic flow chart of early muscle development where the main patterning events have been defined. *h.p.f*. hours post-fertilization, *ABC* anterior border cells, *Row1* row of cells 1

In zebrafish, the first somite forms shortly after the end of gastrulation [84]. The paraxial mesoderm develops from the cells around the edge of the early gastrula, which converge towards the dorsal side to form the paraxial mesoderm, adjacent to the axial mesoderm (Fig. 3). As somitogenesis proceeds, the trunk begins to lift off the yolk and the tail extends. At the end of the first day of development, somitogenesis is complete, and the somites are subdivided into sclerotome and *myotome*, where the myotome is already innervated and functional [84].

One of the most intriguing differences from mammals is that, in zebrafish, the myoblasts become committed to myogenic progenitor cells before the onset of somitogenesis. The first wave of myogenesis comes from a medial *myoD*-positive and *myf5*-positive presomitic mesoderm cell population that lies adjacent to the notochord, known as the *adaxial cells* [85]. After somite formation, these adaxial cells migrate radially from the notochord to form a layer of superficial slow fibers on the myotomal surface that uniquely express the transcription factor Prox1, and slowMyHC (*smyhc1*) [8]. Unlike adaxial cells, the lateral population of cells in the segmental plate, known as the lateral presomitic cells, does not express detectable levels of *myoD* and *myf5* until somite formation. After slow-fiber precursor migration and differentiation, the lateral presomitic cells in the deeper and posterior part of the somite differentiate into medial fast muscle fibers, which then form the second component of the primary myotome [86–88]. At 24 h post-fertilization (hpf), the segmentation is complete and a functional myotome is formed.

After this embryonic period, new muscle fibers differentiate into several body locations in a process called stratified hyperplasia, or secondary myogenesis (48–72 hpf) [8, 89]. Indeed, in teleosts, muscle growth occurs both by hypertrophy, due to the increase in size of pre-existing muscle fibers throughout life [6], and by hyperplasia, due to the activity of myogenic progenitor cells in the larval stage [90]. As in amniotes, continuous growth of the myotome relies on myogenic progenitor cells that originate from the *zebrafish dermomyotome* [7].

The dermomyotome was initially characterized in the late nineteenth century on the basis of anatomical and morphological evidence, and, in teleosts, it has received renewed interest only recently [91]. As in amniotes, the teleost dermomyotome expresses Pax3 and Pax7, although in zebrafish it consists of a flattened epithelium with no obvious dorsal and ventral lips [7, 92]. As the adaxial cells and posterior somite cells express the MRFs very early, are post-mitotic before their incorporation into somites and do not express Pax3 and Pax7, this suggests that the primary myotome develops independently of the dermomyotome [86, 90]. Indeed, interestingly, in zebrafish, the first muscle fibers elongate before the dermomyotome forms, at variance with amniotes, in which the first muscle fibers elongate after the dermomyotome development.

The cells from which the dermomyotome in teleosts originate have been defined as the anterior border cells, to distinguish them from the medial and posterior region of the somite [86]. Moreover, because these *myoD*-negative anterior border cells form a single row of epithelium that is external to the myotome, they have also been called Row1 cells (Fig. 3) [90]. During late segmentation and early larval stages, dermomyotomal cells proliferate and give rise to the secondary myotome: the mesenchyme cells of the dorsal fin, fin muscle, and dermis. As indicated above, the earliest growth of the primary myotome occurs through stratified hyperplasia, which produces layers of fibers with different cross-sectional areas. The dermomyotome Pax7-expressing cells move from the outside surface to the inside surface of the slow muscle monolayer and originate new fast muscle fibers that are initially added in the region between the slow and fast fibers [86, 90]. Interestingly, teleosts retain an epithelial layer of Pax7-undifferentiated-positive cells into their early juvenile period, thus leading to a continuous contribution to post-larval muscle growth. A wide and deep comparative analysis of the zebrafish dermomyotome was provided by Stellabotte and Devoto [91] (Fig. 3).

In zebrafish, it has been reported that either Myf5 or MyoD are sufficient to promote slow muscle formation from adaxial cells, and that MyoD is required for fast muscle differentiation [92, 93]. Indeed, down-regulation of both MyoD and Myf5 abolishes slow muscle development in the early myotome, whereas MyoD, but not Myf5, cooperates with Pbx homeodomain proteins to promote a fast myogenic program [94]. Interestingly, and at variance with mammals, Mrf4 does not control early myogenesis in zebrafish, as Mrf4 is expressed as late as Myogenin, and therefore has no role in muscle commitment [95–97]. Lack of skeletal muscle in the double *myf5:myoD* mutants shows that endogenous zebrafish Mrf4 cannot drive early myogenesis in the *myf5:myoD* double morphants [98], unlike the situation in mouse [26]. Of note, the injection of *mrf4* mRNA, but not *myogenin* mRNA, can rescue and drive myogenesis via the robust activation of endogenous *myoD* [97]. Additionally, zebrafish MyoD can activate *mrf4* in *myf5*-mutant embryos, which indicates a positive-feedback loop between *myoD* and *mrf4* in zebrafish; such a positive-feedback loop has not been reported in any other species to date [97].

The *head musculature* in zebrafish has recently been explored, although there is little evidence available on this topic. Almost all the cranial muscles contain both slow and fast muscle fibers, and, as in the trunk, slow fibers are found only in the periphery of each muscle [99]. Additionally, the proportion between slow and fast muscles varies through development, with a decrease in the proportion of slow muscle with ontogeny. The transcription factor Six1a has an essential role in craniofacial myogenesis, as it is necessary for MyoD and Myogenin expression in the head [100]. Remarkably, and at variance with what is observed in mammals, in zebrafish, MyoD is necessary to drive the cranial musculature, as *myod* morphants show reduced muscle fibers in the head [98]. The mechanisms of action of Six1a in the development of the head muscle were well discussed in Lin et al. [100].

#### Pivotal role of Hedgehog signaling in teleost myogenesis

As described for mammals, cell fate in the somite of zebrafish also depends on signaling factors released by the surrounding tissues, which thus regulate the balance between proliferation and differentiation. Of note, Hedgehog secreted by the notochord and ventral spinal cord has a crucial, and almost unique, role during myogenesis in zebrafish. Three zebrafish mutants, *floating head* (*flh*),* no tail* (*ntl*), and* bozozok* (*boz*), have defects in the notochord, and these show variable deficiencies in early adaxial cells, *myoD* expression, and development of slowMyHC fibers during the first 24 hpf [88]. In support of this evidence, overexpression of *hedgehog* mRNA in wild-type embryos results in a dramatic expansion of slow muscle at the expense of fast muscle. Conversely, defective slow muscle development in notochord mutants can be rescued by re-expression of wild-type *hedgehog* mRNA [88]. Moreover, overexpression of Patched, which inhibits Hedgehog signaling, as well as a series of different zebrafish mutants of the Hedgehog pathway, promotes defects in slow fiber development, thus supporting the crucial and unique role of Hedgehog in determining the slow muscle fate of adaxial cells [101, 102].

**Fig. 4:**
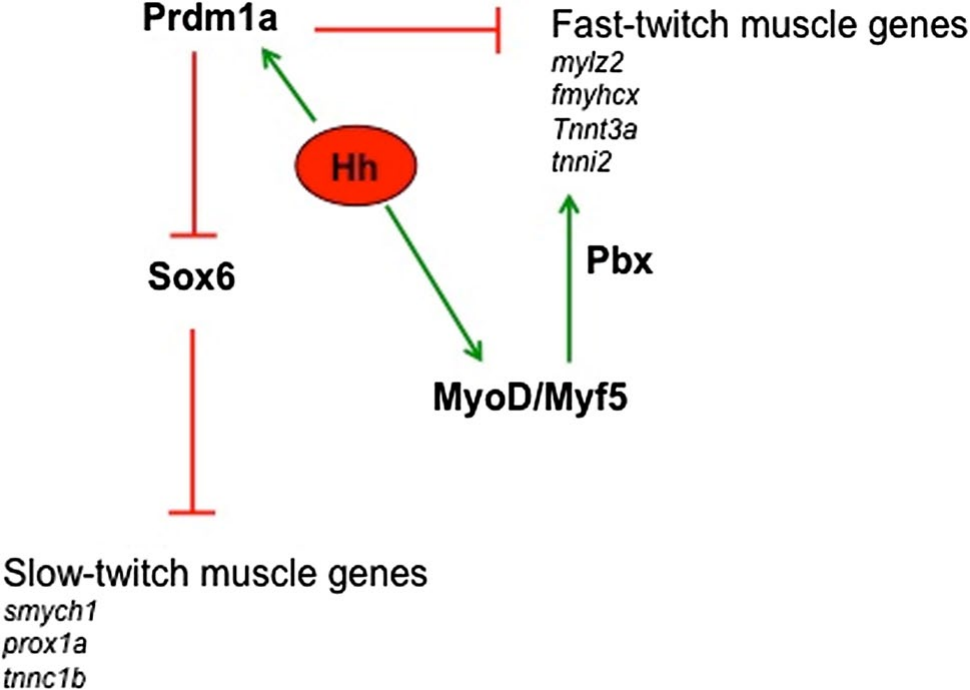
Schematic representation of the molecular pathways that regulate fiber type diversification in zebrafish. In *italics*, specific slow and fast genes; see text for details

Intriguingly, the group of Devoto demonstrated that Hedgehog also has a later role in the regulation of differentiation of the Pax3- and Pax7-positive dermomyotomal population [103]. Using various tools to inhibit the Hedgehog pathway, it has been demonstrated that Hedgehog does not regulate the induction of Pax3 and Pax7 expression in the dermomyotome, but instead affects the down-regulation of Pax3 and Pax7 which is a necessary step for the subsequent expression of *myoD* and for differentiation into fast muscle fibers (Fig. 4). Moreover, it has been shown that the effect of Hedgehog on embryonic slow and fast muscle fibers can be distinguished both pharmacologically and genetically: slow muscle fibers require earlier Hedgehog signaling and are dependent on the downstream Hedgehog effector *gli2*, whereas fast muscle differentiation requires later Hedgehog signaling, and these fibers are only in part dependent on *gli2.* Intriguingly, Feng et al. [103] created genetic mosaics by transplanting either wild-type or Hedgehog-unresponsive *smu(smo)* ^−*/*−^ cells into wild-type embryos, through which they showed that the requirement for Hedgehog signaling is cell autonomous in the dermomyotome and is not mediated by another signal released by the adjacent slow muscle fibers. It is possible that the role of Hedgehog in mammals is more likely to be analogous to the second action of Hedgehog in zebrafish, which is on Pax3- and Pax7-positive dermomyotomal cells.

As in mammals, the activities of TGF-β family members, such as Bmp4, oppose the actions of Hedgehog signaling on adaxial cells [104]. It has recently been observed that even the dermomyotome is responsive to Bmp2b and Bmp4, the actions of which increase the number of Pax7^+^ myogenic progenitors, which thus delays muscle differentiation. Of note, while BMP overexpression is sufficient per se to interfere with terminal differentiation, BMP inhibition does not affect this process, which thus indicates that other factors can redundantly inhibit myogenic differentiation [105]. So, despite important differences in the fate of myoblasts within the somite in mammals and teleosts, the opposite actions of Hedgehog and BMP4 in somite patterning appear to be conserved throughout vertebrate evolution. Little is known or has been explored relating to the role of Wnt signaling in zebrafish myogenesis.

#### Control of muscle fiber type diversity during embryonic development

As indicated above, different fiber type compositions reflect and respond to the different needs of an individual. This diversity is influenced by external stimuli and cues, and it is also finely controlled by signaling pathways during development. As in mammals, even fiber type development and distribution in zebrafish is driven by defined molecular pathways that have been extensively studied in the last 15 years [106].

The studies from the group of Ingham have defined the transcriptional regulatory network at the base of fiber type diversification [106–108]. With Hedgehog signaling added to a slow myogenic program, this network involves the repressor activity of Prdm1a, a zinc-finger DNA-binding protein that can promote the slow-twitch differentiation program by direct inhibition of the fast specific genes, such as *mylz2*, *fastMyHCx*, *tnnt3a*, and *tnni2*. In contrast, Prdm1 appears not to bind promoters of typical slow genes, such as *prox1a*, *smyhc1*, and *slow troponin c*, and functions instead by repression of the transcription factor *sox6* [107]. As in mammals, Sox6 in zebrafish is expressed in fast-twitch progenitors, and it can repress slow-twitch genes [79, 106–108]. Recently, Wang et al. defined a gene regulatory network that shows temporal control of the slow-specific program through a post-transcriptional feedback circuit that involves the activity of miR-499. As in mammals, miR-499 arises from an intronic region of the *slowMyHC* gene and specifically inhibits *sox6* [108] (Fig. 4). Intriguingly, and at variance with what has been observed in mouse, the loss of Sox6 in zebrafish does not lead to ectopic expression of all the slow genes in the fast fibers, as *smyhc1* gene expression remains confined to slow muscle fibers [107].

Although there has been *sox6* gene duplication in most teleost genomes, this duplication did not occur in zebrafish [106]. This leads to the assumption of another repressor that is potentially involved in the repression of *smyhc1* in non-adaxial cells.

Although the majority of the fish musculature comprises fast-twitching myofibers, less is known or has been investigated relating to the signaling responsible for fast-muscle specification. In zebrafish, Six1 and, most importantly, the Pbx homeodomain transcription factors have been implicated in the control of the onset of fast-muscle differentiation. In particular, it was recently demonstrated that Pbx acts by directing MyoD to a subset of fast-muscle genes, which counteracts the repressing action of Prdm1a [94, 109, 110].

To date, different aspects diversify fiber type determination in mammals and zebrafish. First, while in zebrafish the adaxial cells give rise to only slow muscle [8], in amniotes, the slow myogenic program is not determined by a specific myogenic population. Slow and fast muscle fibers can both arise from the same cell type, which only depends on the determination signals that they receive. Secondly, in mammals, slow and fast myofibers are both multinucleated, although they differ in muscle size [66], whereas in fish, the slow-twitch progenitors are fusion incompetent and differentiate into mononucleated fibers, at variance with their fast-twitch myoblasts [8, 111]. In addition, although Prdm1 is highly conserved among vertebrates and its expression is dependent on Hedgehog signaling, the pivotal role of Prdm1a in the regulation of slow muscle differentiation in zebrafish is not conserved in mammals, in which its absence does not impair correct fiber type determination and Sox6 expression [106, 112].

#### Myogenic waves in zebrafish

We have already indicated that amniotes have multiple, distinguishable waves of myogenic differentiation during pre-natal muscle development, and that these myogenic waves tightly depend on defined embryonic and fetal myogenic populations that share distinct genetic programs [66, 67, 73]. In teleosts, the boundaries are less defined, and the main differences arise because different myogenic populations promote distinct myogenic programs that are defined on the basis of the positions where these populations will form muscle fibers. At the end of the segmentation period in zebrafish (24 hpf), the events that lead to a functional embryonic myotome can be defined as the *primary myogenic wave*, the timing and characteristics of which cannot be directly compared to mouse embryonic/primary myogenesis. Following this primary myogenic development, the secondary muscle fibers differentiate in several body locations in a process called stratified hyperplasia or *secondary myogenesis* (48–72 hpf) [6, 8, 89]. This is more similar to mouse primary/secondary myogenesis on the basis of the source of the myogenic populations (the dermomyotome), the requirement for Hedgehog signaling, and the formation of multinucleated slowMyHC. Nevertheless, a convincing comparison is still difficult. As indicated above, we identified the transcription factor Nfix as a master switch regulator of the transcriptional transition from embryonic to fetal muscle [73]. According to these data, if Nfix has a similar role in zebrafish, this would allow better definition of the myogenic waves in teleosts. We demonstrated that there is a zebrafish ortholog of Nfix, *nfixa*, the mRNA of which strongly increases at the onset of the secondary myogenic wave, as in mammals [113]. Indeed, using a loss-of-function approach to specifically abrogate the *nfixa* function in vivo, we showed that lack of *nfixa* does not perturb the primary myogenic wave, as the injected embryos showed normal somite numbers and size, normal MRF expression, and normal superficial slow fiber formation. In contrast, this loss of *nfixa* led to effects that were evident from 48 hpf that caused a marked impairment of the second myogenic wave: embryo immobility, persistence of *smyhc1* expression, lack of a Pax7^+^ population, and muscle disorganization. As in mammals, Nfixa acts through conserved mechanisms that include *nfatc4*-mediated regulation of slowMyhC expression and cooperation with the Mef2 proteins [113]. Of note, we also observed that the *nfixa*-morphants did not move and swim correctly due to impaired development of the sarcoplasmic reticulum, which was not observed in *Nfix*-null fetuses [73, 113]. Therefore, although the mechanisms underlying the second myogenic wave in zebrafish have been poorly characterized, our data shed light on the conserved functions of Nfix in this process, and on this first comparison between the second myogenic wave in zebrafish and fetal myogenesis in mammals.

### Post-natal myogenesis in mammals

#### Amniote satellite cell properties

**Fig. 5:**
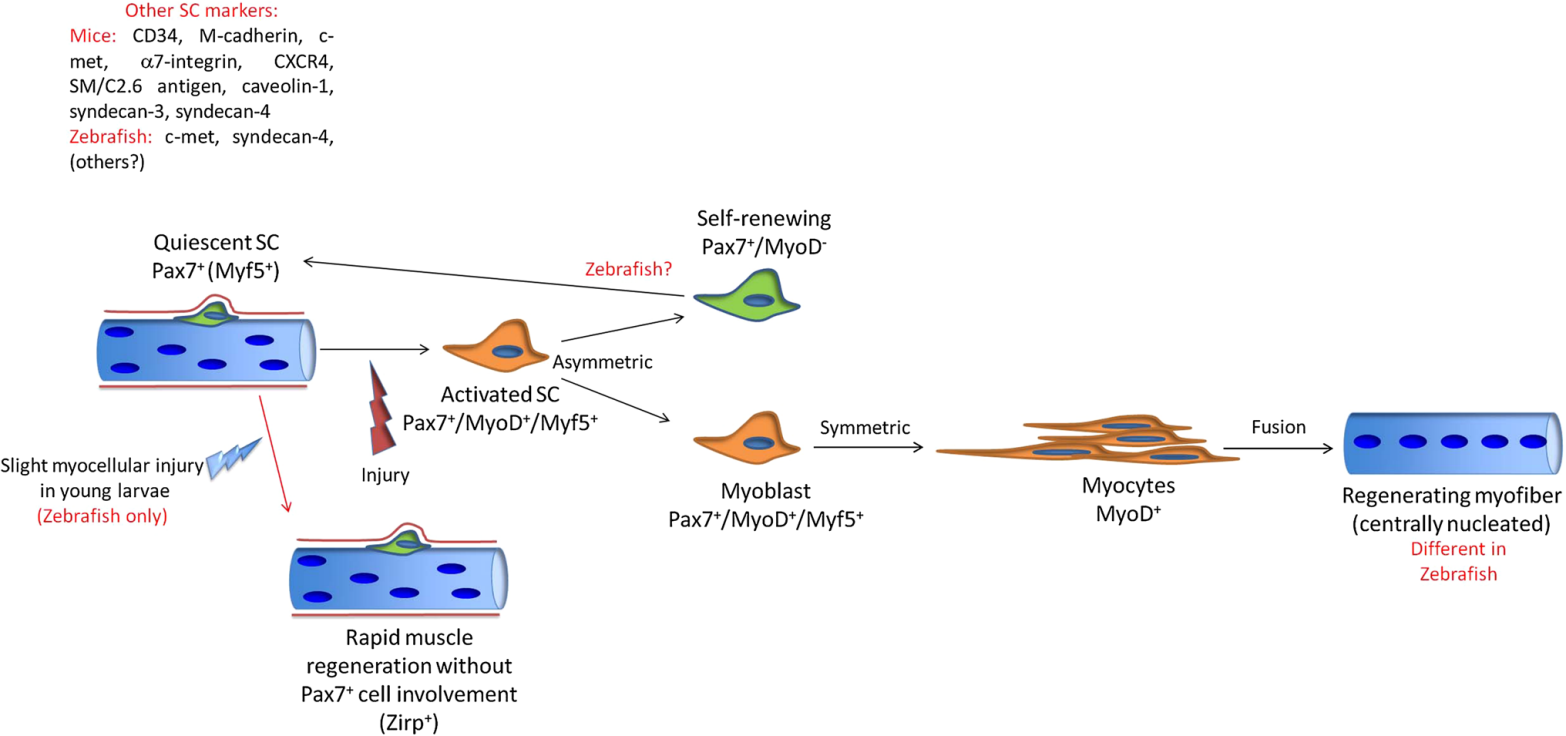
Comparative scheme of regenerative myogenesis in mammals and teleosts. *Red* highlights the main differences

Post-natal skeletal myogenesis in mammals mainly relies on SCs, which are adult muscle-resident stem cells that are responsible not only for post-natal muscle growth but also for muscle regeneration after damage. These cells were first identified in 1961 by Mauro [114], who named them for their ‘satellite’ position with respect to the myofiber. Indeed, starting from E16.5 in mouse, SCs can be easily identified using electron microscopy as mononucleated cells positioned at the periphery of myofibers, between the basal lamina and the sarcolemma (Fig. 5).

During the first 3–4 weeks of post-natal life in mouse, juvenile and actively proliferating SCs are responsible for perinatal muscle growth, while successively, once the adult has reached a fixed body size, their SCs remain in a G0 phase until they are correctly stimulated [68]. Muscle and body size can be further regulated by the modulation of specific signaling pathways, such as overexpression of the insulin-like growth factors [115] or repression of Myostatin (GDF-8), a TGF-β family member, which is a well-known myogenesis inhibitor [116].

Although the adult SC population accounts for less than 5 % of the total number of nuclei, when there is muscle damage, these cells can re-enter the cell cycle, and rapidly proliferate and differentiate into new fibers. This property explains the ability of skeletal muscle to extensively regenerate, even if the mammalian myonuclei are post-mitotic [117, 118].

To maintain a quiescent pool of SCs through multiple regenerative cycles, SCs have been shown to undergo asymmetric cell division, which gives rise to one daughter cell destined to self-renew and another committed to differentiation [119–123]. According to the ‘immortal DNA strand hypothesis’ [124], the occurrence of asymmetric divisions ensures the co-segregation of both parental DNA strands into the self-renewing daughter cell (a process that is also referred to as ‘template DNA strand segregation’). This process therefore avoids the accumulation of mutations during replication in the stem cell population. In vivo self-renewal of SCs has been demonstrated by transplantation experiments. Due to the difficulties in obtaining a pure, still quiescent population of dissociated single SCs, self-renewal was initially demonstrated by transplantation of an entire myofiber in irradiated mice [125]. The more recent advances in SC isolation techniques have provided verification of in vivo self-renewal from single transplanted SCs [126], and have demonstrated that SCs retain stem cell function over multiple rounds of serial transplantation [127]. SCs actually represent a heterogeneous population in terms of their developmental origin, and they show expression of different markers, and have different growth factor requirements, structural gene expression upon differentiation, and stemness properties [128].

#### Satellite cell origin and markers

The SCs of the limb and back muscles share a common somitic origin [129–131], while the SCs in the head muscles derive from prechordal and cranial paraxial mesoderm [132]. This suggests that SCs reflect the different embryonic origins of the muscle in which they reside. Due to the greater number of studies of the limb musculature, this review will mainly focus on post-natal myogenesis of SCs in the limb muscles.

Adult muscle progenitors in the limbs originate from multipotent cells in the dermomyotome that express Pax3 and Pax7. This population represents a reservoir of muscle-resident progenitor cells that continue to proliferate during embryonic and fetal development, where they contribute to muscle development, and then adopt the SC positioning during post-natal life [43, 53]. Recently, it was demonstrated that SC progenitors are also primed by Myf5 [133], Mrf4 [134], and MyoD [135, 136] during prenatal phases. During adulthood, Pax3 expression is restricted to distinct muscles. In particular, there are Pax3-positive SCs in the diaphragm and in some forelimb muscles, while they are almost absent in the hindlimbs [137]. In contrast, Pax7 is expressed in all quiescent SCs and activated myoblasts, and it represents the main marker of SC populations. Indeed, high levels of Pax7 expression have been shown to mark the quiescent or ‘stem’ state of SCs [120, 127, 138]. Pax7 has been shown to be necessary for maintenance of SCs during perinatal and juvenile life. Indeed, *Pax7*-null mice have skeletal muscle deficiency at birth, which suggests a unique requirement for Pax7 in myogenic SC function. In these mice, SCs are present at birth and can differentiate into skeletal muscle, but they are progressively lost during post-natal development [52, 137, 139, 140]. In 2009, Lepper et al. [141] used tamoxifen-induced *Pax7* inactivation to demonstrate that, when *Pax7* is inactivated in adult mice, SC function is not compromised, and regeneration occurs correctly. Very recently, however, their study was reinterpreted, as it has been demonstrated that continuous tamoxifen administration with chow results in sustained *Pax7* deletion that finally leads to defective muscle regeneration, due to cell-cycle arrest and precocious differentiation [142, 143], therefore also underlining the main role for Pax7 in adult SCs.

Other well-known SC markers include CD34 [144], M-cadherin [145, 146], c-met [145], α7-integrin [147, 148], CXCR4 [149], SM/C2.6 antigen [150], Caveolin-1 [147, 151], and Syndecan-3 and Syndecan-4 [152, 153].

As myonuclei are post-mitotic, muscle regeneration after damage relies completely on SC activation, proliferation, withdrawal from cell cycle, terminal differentiation, and fusion together (for de novo myotube formation) or with damaged myofibers (to replace lost myonuclei). These processes partially parallel developmental myogenesis, in which fusion of mononucleated muscle progenitors gives rise to multinucleated muscle fibers. Similarly to what happens during prenatal muscle development, post-natal myogenesis that is driven by SCs follows a precise regulatory factor hierarchy, with the MRFs acting downstream of the *Pax* genes.

#### Initial steps of muscle regeneration: inflammatory phase and satellite cell activation

Adult, Pax7-positive SCs are quiescent and in G0 during homeostasis, with the *Myf5* locus already active in many quiescent SCs [144, 154]. Myf5 is considered to be the first marker of myogenic commitment, and, therefore, to justify the positivity of some quiescent SCs for Myf5, it has been proposed that many SCs become quiescent after committing to skeletal muscle lineage [122]. According to this theory, the population that is negative for Myf5 would represent the stem cell compartment that is destined for self-renewal. To explain how SCs can maintain quiescence while already being primed for differentiation, it has recently been proposed that Myf5 mRNA in quiescent cells is sequestered in mRNP granules, and only released upon activation [155]. As further support for this, it has also been proposed that Myf5 protein levels regulate muscle stem cell fate by regulating the balance between commitment and self-renewal [156].

As a consequence of muscle injury or in chronic diseases, skeletal muscle can degenerate, and the following regeneration process can be divided into three different phases: inflammation, tissue reconstruction, and tissue remodeling. Inflammation occurs after plasma membrane disruption and the consequent chemotactic recruitment of inflammatory cells. The first inflammatory wave is mainly composed of neutrophils, then a major role is played by macrophages, initially with phagocytosis of the cellular debris in the necrotic area, and then through sustaining SC proliferation and differentiation [157]. Already during the initial inflammatory phase, SCs are activated to successively undergo rapid proliferation, followed by differentiation and fusion with existing myofibers or with other myoblasts. During the proliferative phase of muscle reconstruction, newly activated SCs re-enter the cell cycle, start to proliferate, and co-express Pax7 and MyoD, which is the hallmark of the activated state [138, 158, 159]. Interestingly, while most SCs undergo proliferation and down-regulate Pax7 before differentiation, some retain Pax7 expression, down-regulate MyoD, and return to quiescence [138, 159]. MyoD also appears to have a role in the regulation of the balance between self-renewal and differentiation, as *Myod* ^−*/*−^ adult mice have increased numbers of SCs and myoblasts [160, 161]. Myf5 also has a role during differentiation, whereby *Myf5*-null mice are characterized by delayed regeneration, with the formation of hypertrophic myofibers and the persistence of fibrosis [156].

#### Satellite cell differentiation and tissue remodeling

After proliferation and migration through the damaged area, early SC differentiation leads to the expression of Mrf4 and Myogenin, along with transcription factors of the MEF2 family. Both Mrf4 and Myogenin are only expressed during differentiation [159, 162, 163], and they appear not to be necessary for adult muscle progenitors [164–166]. Terminal differentiation is marked by the expression of sarcomeric and sarcoplasmic proteins and by fusion into multinucleated myotubes. During regeneration, typical developmental markers are re-expressed, such as embryonic and neonatal MyHC [125, 167, 168]. The expression of these MyHC isoforms in adult mice is considered to be a marker of ongoing regeneration. SC differentiation is followed by fusion and maturation of the regenerating myofibers, which are initially characterized by a reduced cross-sectional area and by the presence of centrally located myonuclei. The extent of tissue remodeling depends on the extent of damage and on the involvement of the basal lamina. Maturation and tissue remodeling require the re-establishment of the neuromuscular junctions and the expression of adult MyHC isoforms, as the initially fast and then also the slow [169, 170]. In adult mice, the entire process of muscle regeneration upon acute injury is completed within 3–4 weeks [68].

#### Aged satellite cells and unorthodox myogenic populations

With age, the number of SCs in skeletal muscle progressively decreases [171, 172]. Intriguingly, however, the intrinsic regeneration potential is maintained through time, as demonstrated by the successful regeneration that has been obtained after grafts of old muscle cells into young hosts [173, 174], and by parabiosis experiments where young and old SCs share the same microenvironment [175]. This evidence strongly suggests that the niche microenvironment has a central role in determining the SC potential.

As well as SCs, other unorthodox myogenic populations have been identified that can generate skeletal muscle both in vivo and in vitro [148, 176–183]. However, recent studies using inducible genetic strategies to ablate adult Pax7^+^ SCs have demonstrated that, in the absence of SCs, skeletal muscle repair after damage is not successful, thus demonstrating that the in vivo contribution of these alternative cell types is secondary to the major role of the SCs [184–187].

### Post-embryonic myogenesis and regeneration in zebrafish

#### Post-larval muscle growth

At variance with mammals, where muscle and body growth are determined and finalized according to a fixed body size, many fish show indeterminate growth through both hyperplasia and hypertrophy mechanisms in response to multiple factors, thus providing continuous growth throughout their entire life [188]. Interestingly, however, muscle growth in zebrafish is determinate, with very little muscle fiber hyperplasia after the juvenile phase, even when subjected to growth hormone treatment [189]. Similar to mammals [116, 190], in zebrafish, inhibition of Myostatin leads to enhanced hyperplasia and, in some cases, hypertrophy, and consequently to increased body size [191–193]. This evidence highlights a conserved role for Myostatin in the regulation of muscle growth. However, while expression of Myostatin in mice is mainly confined to skeletal muscle, in fish, Myostatin is expressed in a variety of other tissues [194, 195], which suggests possible nonconserved roles between mammals and teleosts.

#### Muscle regeneration properties and first satellite cell identification

In past years, it was generally believed that zebrafish larvae can regenerate muscle tissue through a de-differentiation process, similar to that of many amphibians. However, a recent study has shed particular light on this aspect, with the confirmation of the absence of epimorphic skeletal muscle regeneration in zebrafish larvae [196]. Thus, it is now, in contrast, universally accepted that teleosts can generate and regenerate muscle from a population of ‘satellite-like’ myogenic precursor cells. As in mammals, in zebrafish, mitotically inactive Pax7^+^ cells that originate from the dermomyotome can be identified beneath the basal lamina of the myofiber throughout the larval, juvenile, and adult stages [90, 197]. Their quiescent state, position, expression of Pax7, and permanence in the adult stages have suggested that they represent a resident progenitor cell population similar to mammalian SCs. Moreover, these Pax7^+^ cells in zebrafish also express other typical SC markers, such as the HGF receptor c-met [90] and Syndecan-4 [198].

However, evidence for rapid recovery from slight myocellular injury within the zebrafish embryo without the involvement of cell proliferation has recently been reported. These events have been associated with positivity for Xirp (Xin-actin-binding repeat-containing protein) in the damaged area [199], which represents a unique feature with respect to mammals. Interestingly, however, the Xin proteins have also been associated with muscle regeneration in mice [200].

The regenerative ability of zebrafish muscles upon acute injury and in chronic pathologies has also been characterized [197, 201]. In particular, the recent generation of a *pax7a*-reporter zebrafish line allowed the behavior of Pax7-expressing cells after cardiotoxin injury and in dystrophic models to be follow precisely [197]. Similar to mammals, muscle injury in teleosts results in muscle progenitor cell migration to the site of injury and proliferation, as evident from the marked increase in BrdU incorporation at the early stages following muscle damage, along with the degeneration and necrosis of the damaged muscle fibers. At the same time, expression of the MRFs is strongly up-regulated in myofibers near the site of injury, in order to sustain the process of regeneration [197, 201]. As in mammals, damaged tissue is temporarily substituted by connective tissue, and then finally newly formed myofibers are identifiable due to their small diameter [197, 201]. One of the most curious differences during regeneration is that, at variance with mice, in which one of the main hallmarks of ongoing regeneration is the presence of centrally nucleated myofibers, central nuclei are only rarely observed during muscle regeneration in teleosts [201]. Intriguingly, regenerating dystrophic zebrafish larvae show reduced levels of central nucleation when compared to wild-type siblings [202], which again shows a strong difference with what happens in mammals, probably due to the different mechanisms that characterize muscle growth in these two models. A comparative model for zebrafish and mammal muscle regeneration is illustrated in Fig. 5.

## Concluding remarks

In the last few decades, zebrafish have emerged as a useful and interesting model for studies on different biological processes that have been widely discussed for higher vertebrates. During this time, it has been demonstrated that a number of developmental events described for mammals are only partially conserved in teleosts. The existence of myogenic progenitors, such as the adaxial cells, the differentiation of a functional myotome before the end of somitogenesis, and the different timing and requirements of the MRFs, clearly demonstrate that direct comparisons between teleosts and mammals are not always possible. Conversely, common features between mammals and teleosts have been extensively demonstrated, such as the source of myogenic progenitors, the molecules involved in myogenic commitment, and the regulation of fiber type diversification.

The possible evolutionary origins of the differences in myogenesis between mammals and teleosts require much more detailed consideration than that behind the scope of the present review. Overall, the existence of common and divergent aspects between myogenesis in mammals and teleosts should be firmly considered during any direct, and potentially forced, parallels between what occurs in teleosts and in mammals. For the muscle regeneration processes, the evidence obtained to date remains too preliminary and not always conserved enough for strong conclusions to be drawn, thus leaving many questions still open that need to be addressed in the future.
